# Skull Oligometastasis From Cervical Cancer: A Case Report

**DOI:** 10.7759/cureus.78115

**Published:** 2025-01-28

**Authors:** Safaa M Alshakankery, Tawfeeq A Tawfeeq, Husam S Almuhaish, Sultan Alsaiari, Mahmoud I Hadad, Marwah Abdulkader

**Affiliations:** 1 Radiology, King Fahad Specialist Hospital, Dammam, SAU; 2 Gynecology, King Fahad Specialist Hospital, Dammam, SAU; 3 Neurosurgery, King Fahad Specialist Hospital, Dammam, SAU; 4 Pathology, King Fahad Specialist Hospital, Dammam, SAU

**Keywords:** cervical cancer, cervical carcinoma, mri brain, skull metastasis, solitary bone metastasis

## Abstract

Bone metastasis is common, mostly resulting from hematogenous spread and usually carrying significant morbidity. The clinical presentation of bone metastasis depends on the site of metastasis and the involved structures. Radiological studies are usually able to detect the location and extent of the lesions with high accuracy. Management of bone metastasis is often a challenging mission. Here, we highlight the case of a 46-year-old postmenopausal lady who was diagnosed with cervical squamous cell carcinoma (SCC), human papillomavirus (HPV)-associated, and International Federation of Gynecology and Obstetrics (FIGO) stage IIIC1, after she presented with postcoital and irregular vaginal bleeding. The patient received chemoradiotherapy. Two months following treatment completion, she complained of painful occipital swelling. Computerized tomography (CT) of the brain revealed an occipital bone lytic lesion without intraparenchymal involvement, which was confirmed by magnetic resonance imaging (MRI). positron emission tomography-computed tomography (PET-CT) and nuclear bone scans excluded other sites of recurrences. She underwent surgical resection for this occipital lesion. The pathological analysis has confirmed the diagnosis of metastatic SCC of cervical origin. Given the poor prognosis of cervical cancer with bone metastasis, early diagnosis and proper palliative treatment probably preserve the quality of the patient’s life despite the expected poor survival rate.

## Introduction

Cervical cancer is the fourth most common cancer in women worldwide, with around 660,000 new cases and around 350,000 deaths in 2022 [1]. In Saudi Arabia, cervical cancer has been reported as the ninth most common cancer among females in childbearing age [2].

Nearly all cases of cervical cancer result from infection with the human papillomavirus (HPV) [3]. About 90% of cervical cancers occur in low-income nations due to unavailable screening or HPV vaccination, as well as poor management facilities for diagnosis [4]. It has been reported that 13% of cervical cancer patients are diagnosed at advanced stages [5].

According to Saudi Arabia's cervical cancer profile published by the World Health Organization (WHO), in 2020, the crude cervical cancer incidence per 100,000 women is 2.4, and the cervical cancer mortality-to-incidence ratio is 0.5 [6].

Cervical cancer is considered a preventable disease. In 2020, the WHO adopted a global plan for the elimination of cervical cancer by 2030 through a 90-70-90 strategy that includes three goals, namely, vaccination for 90% of teenage females, screening for 70% of middle-aged females, and treatment for 90% of females with precancerous conditions or invasive cancer who are receiving treatment [1].

Squamous cell carcinoma (SCC) and adenocarcinoma are the most common histological classifications; moreover, SCC is the widespread subtype, reaching up to 70% of cases [4]. While adenocarcinoma has a worse prognosis compared to SCC [7].

The five-year survival rate of cervical cancer depends on multiple factors, such as the stage of the tumor at the time of diagnosis, age, general health, histopathology type, immunity status, and if the cancer is newly diagnosed or a recurrence [8]. According to the United States National Cancer Institute, the five-year survival rate for early-stage cervical cancer reaches 91.5%, which declines to 16.5% in late-stage cervical cancer [5]. In addition, the site of metastasis is an important factor in assessing the overall survival [9].

Symptoms of metastatic cervical cancer are widely varied according to the site and extension of the metastasis and can include bone pain, pathological fracture, physical impairment, and neurological deficits [10]. The differential diagnosis of bone metastasis includes primary bone tumors such as chondrosarcoma, bone lymphoma, osteomyelitis, and post-radiation sarcoma [10].

In our case study, we report an isolated skull metastasis as a rare site of oligometastasis.

## Case presentation

**Figure 1:**
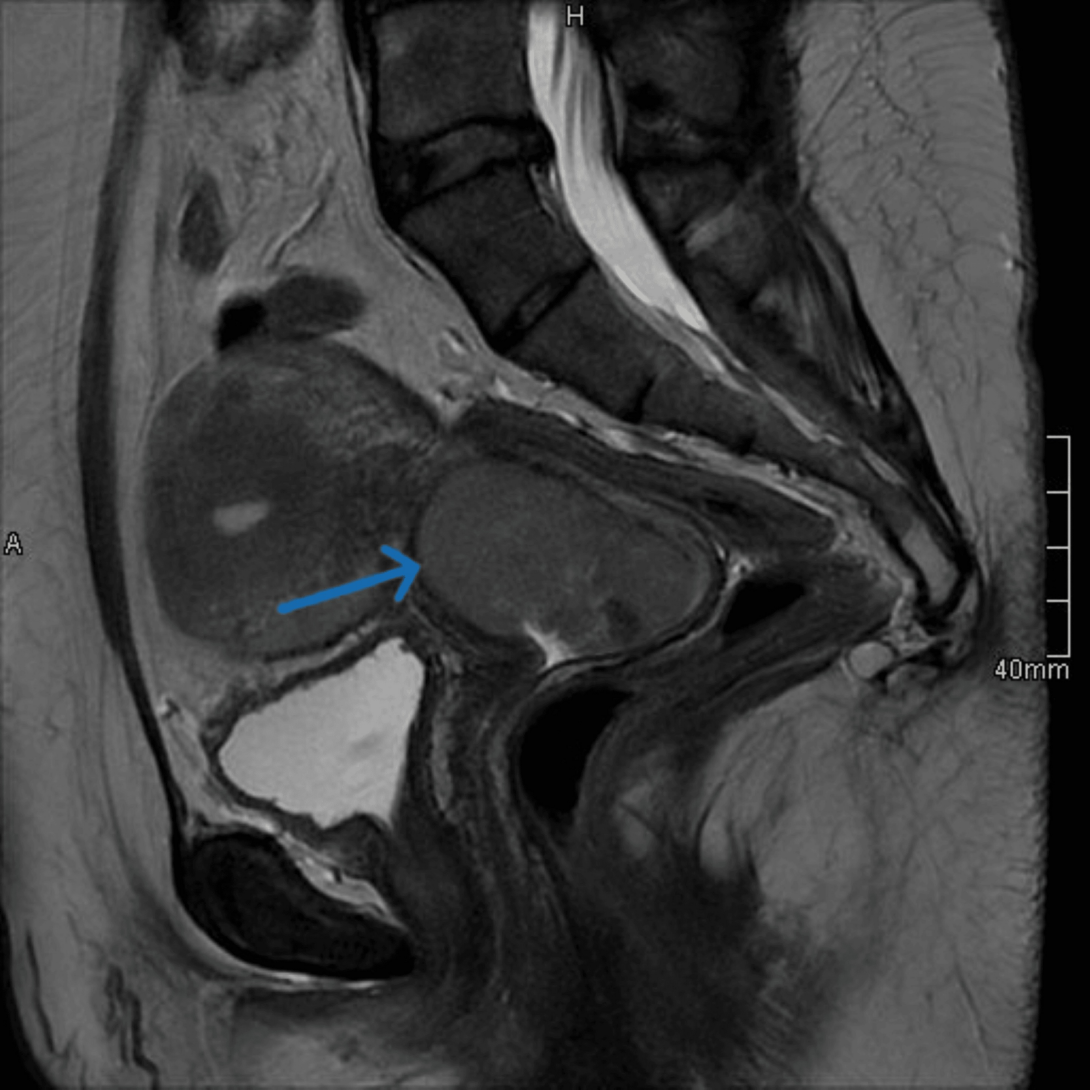
Sagittal T2 MRI of the pelvis demonstrates a large cervical soft tissue mass.

**Figure 2:**
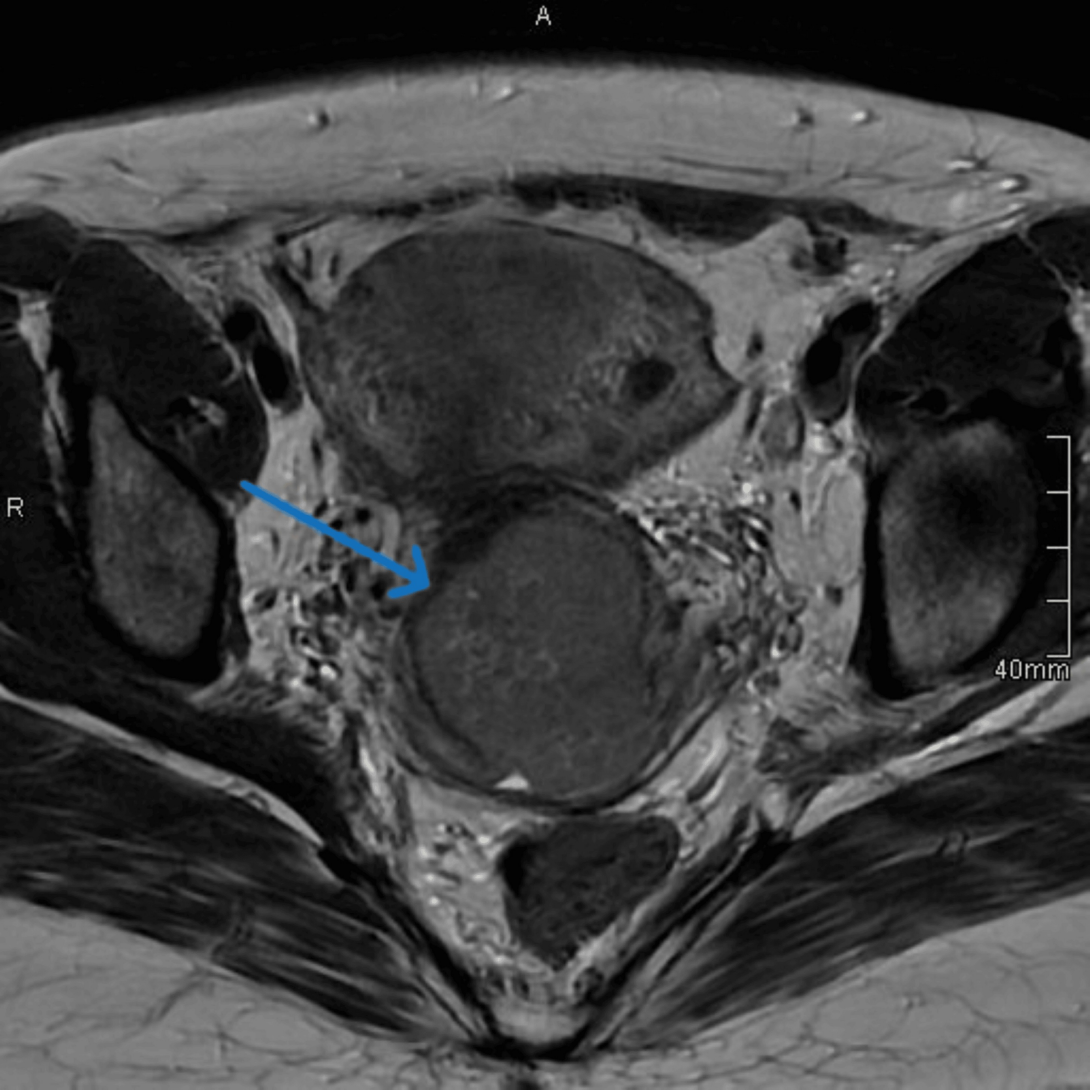
Axial T2 MRI of the pelvis demonstrates a large cervical mass with parametrial invasion.

A 46-year-old lady presented with intermenstrual and postcoital bleeding. The Pap smear revealed epithelial cell abnormality and atypical endocervical cells favoring neoplasia. On pelvic examination, colposcopy revealed an exophytic cervical mass obliterating the cervical os. A biopsy of this mass showed an HPV-associated moderately differentiated SCC. Magnetic resonance imaging (MRI) of the pelvis showed a 5.2 cm cervical mass with parametrial and upper vaginal invasion (Figures 1, 2); the radiological staging was International Federation of Gynecology and Obstetrics (FIGO) stage IIIC1.

**Figure 3:**
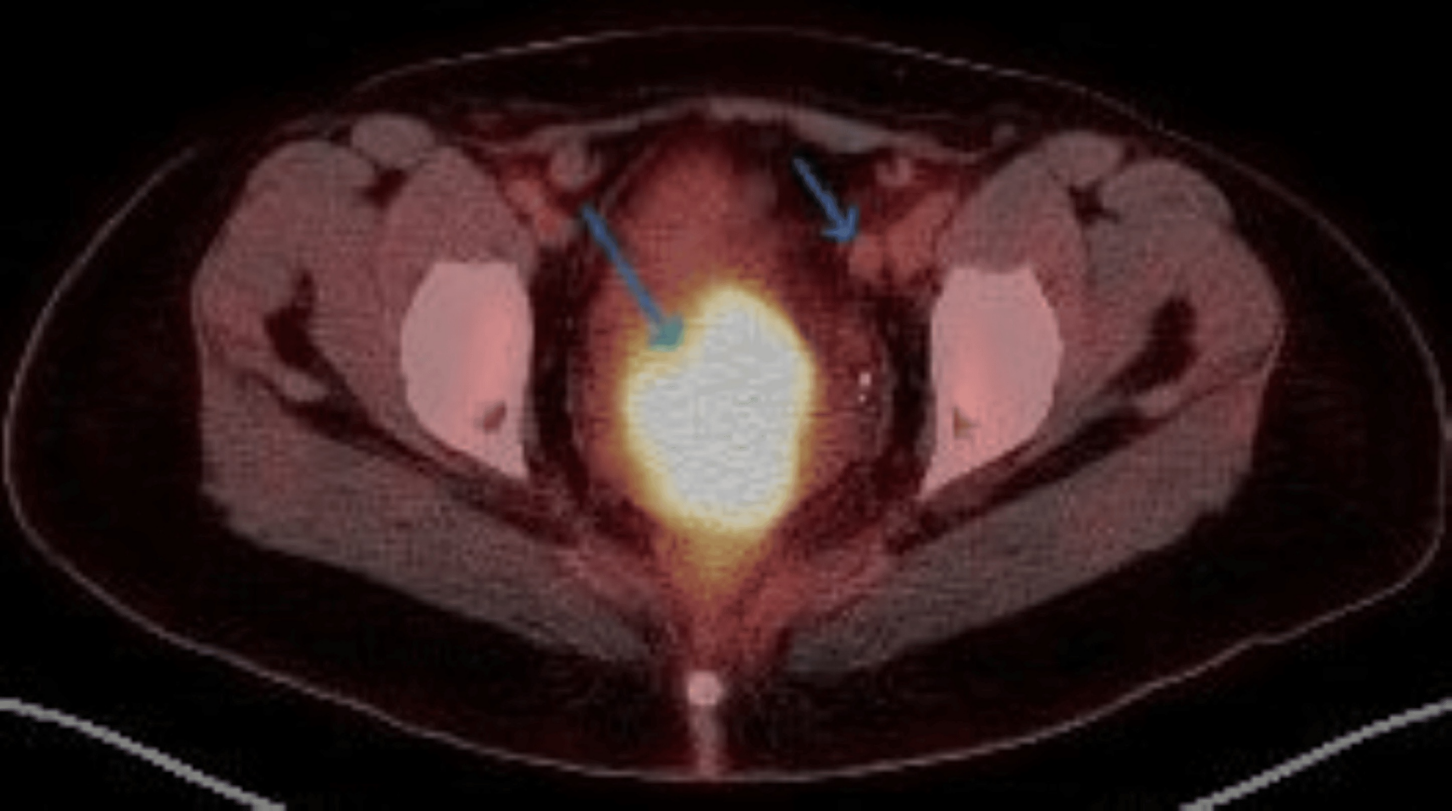
FDG PET-CT demonstrates a strongly avid cervical mass and a mildly avid left external iliac lymph node. FGD: fluorodeoxyglucose; PET-CT: positron emission tomography-computed tomography

Conventional computed tomography (CT) and whole-body positron emission tomography-computed tomography (PET-CT) showed a cervical mass with intense avid uptake and a mildly avid left external iliac lymph node (Figure 3) with no evidence of distant metastasis.

**Figure 4:**
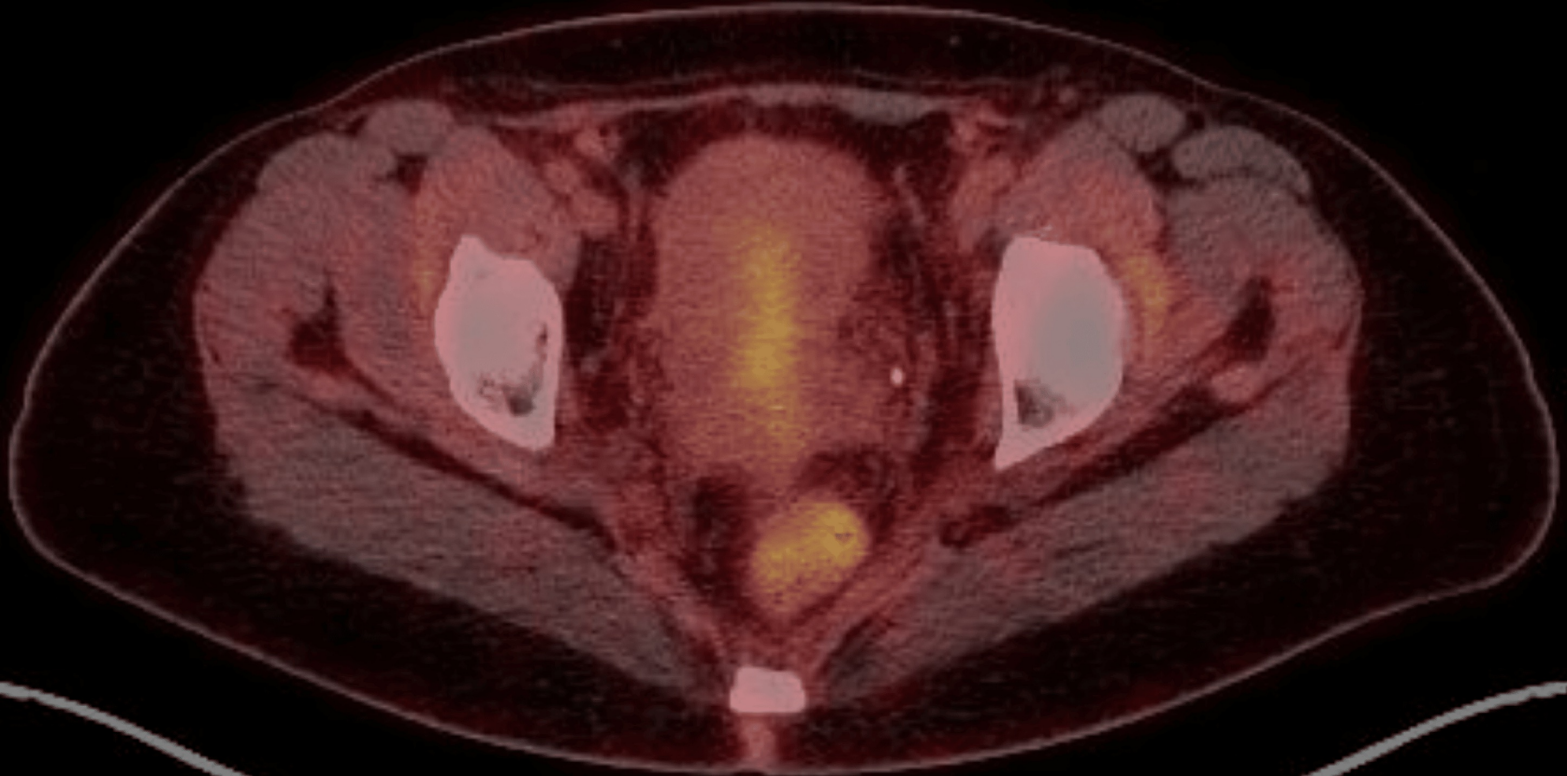
FDG PET-CT demonstrates significant regression of the cervical mass post-CCRT with regard to size and avidity as well as the left external iliac lymph node. FGD: fluorodeoxyglucose; PET-CT: positron emission tomography-computed tomography; CCRT: concurrent chemo-radiation

After a discussion in the multidisciplinary tumor board, all members agreed to offer the patient concurrent chemoradiation (CCRT). Two months following the completion of CCRT, the patient noticed an occipital swelling for three weeks associated with headache. Clinical evaluation has excluded any associated neurological symptoms or signs. A PET-CT was done, which revealed significant regression of the cervical mass and left external iliac lymph node (Figure 4) without evidence of metastasis.

**Figure 5:**
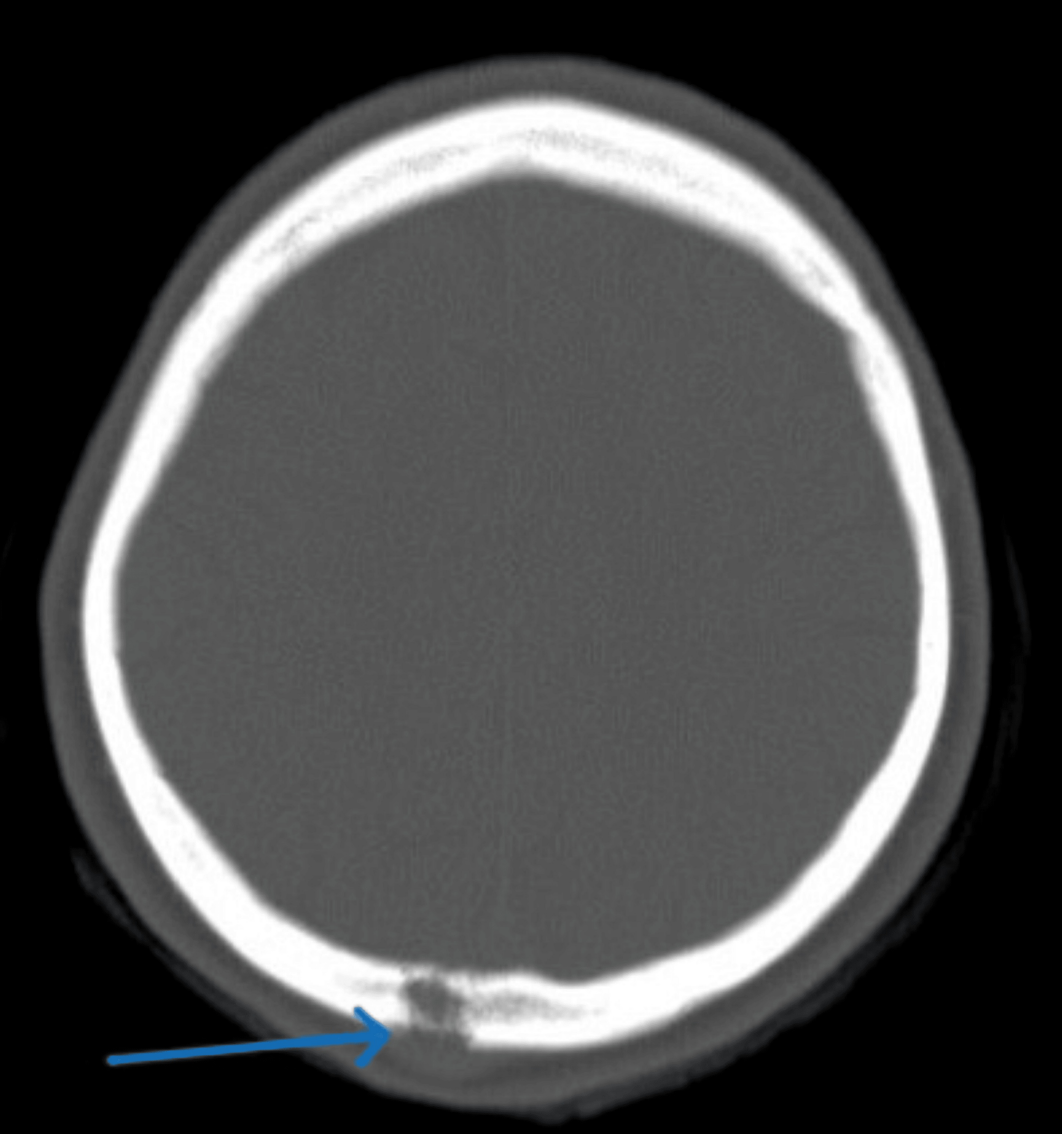
A CT scan of the brain bone window demonstrates a lytic lesion in the occipital bone.

**Figure 6:**
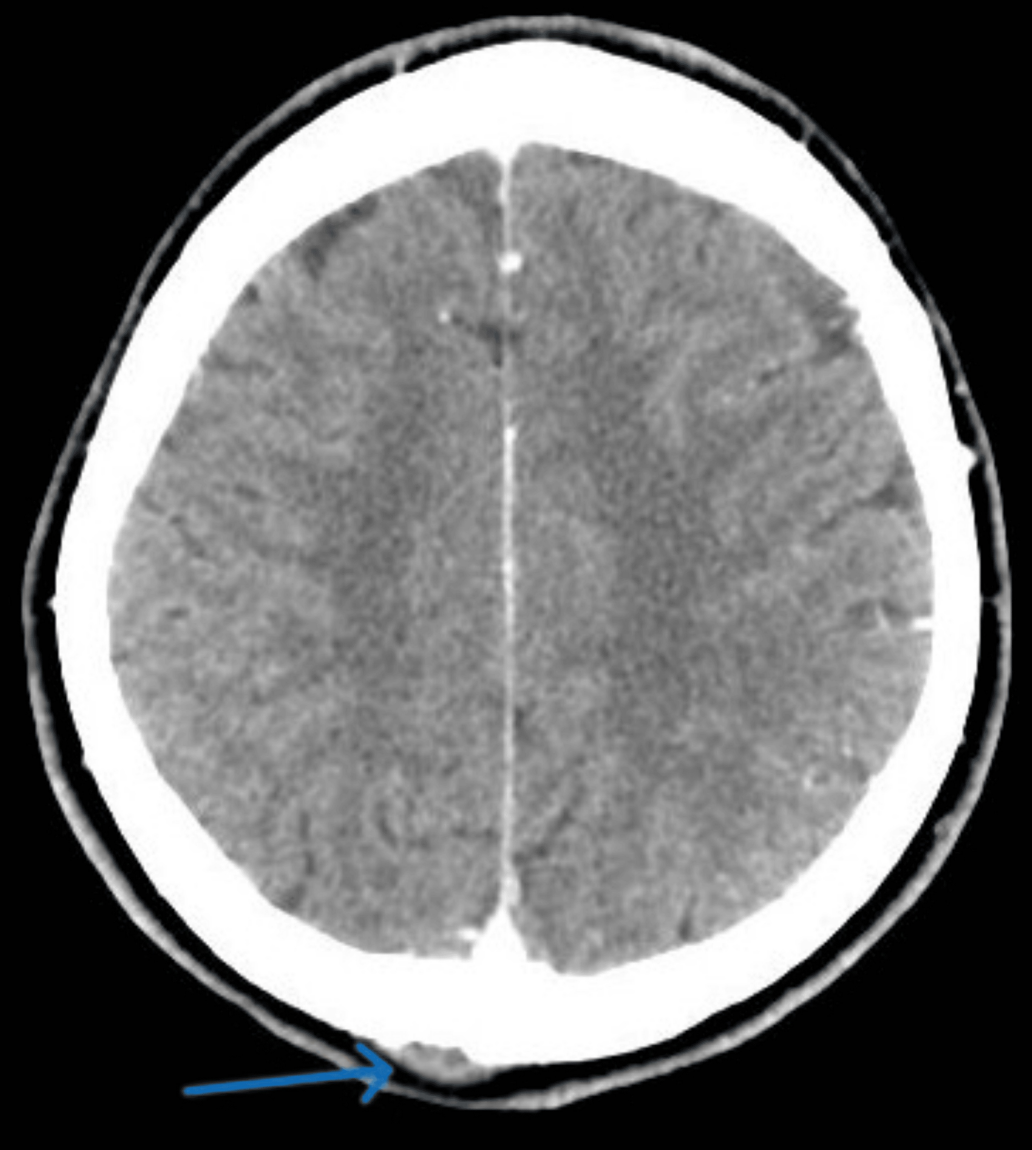
An axial CT scan of the brain with contrast demonstrates a lytic lesion in the occipital bone with a small extracranial soft tissue component.

A CT scan of the brain was carried out which revealed an occipital bone lytic lesion without an intraparenchymal lesion (Figures 5,6).

**Figure 7:**
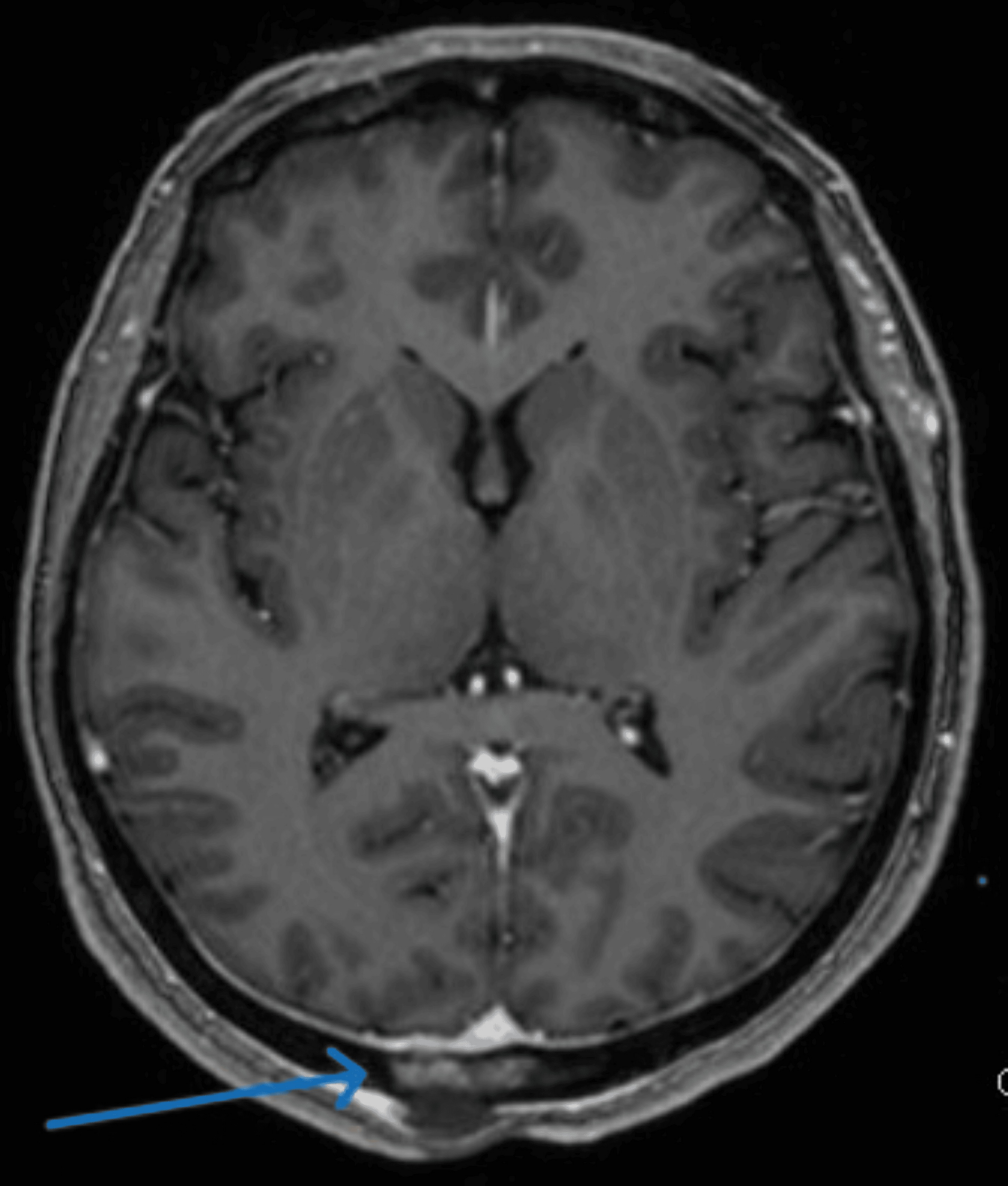
Enhanced axial T1 MRI of the brain demonstrates a right para-midline occipital bone-enhancing lesion with minimal intracranial soft tissue component. No evidence of intraparenchymal lesion or invasion of the venous sinus is noted.

**Figure 8:**
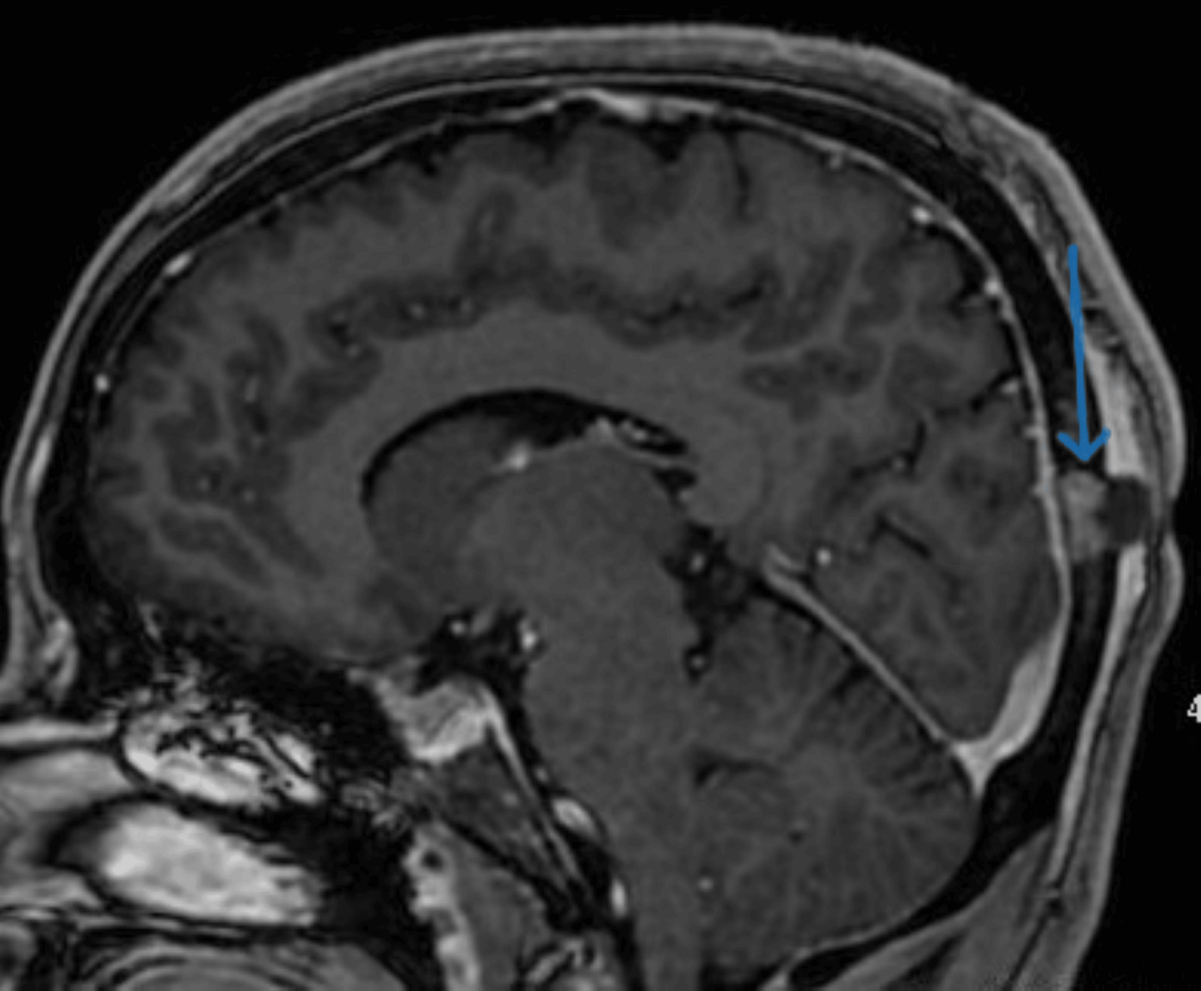
Enhanced sagittal T1 MRI of the brain demonstrates an occipital bone-enhancing lesion with minimal intracranial soft tissue component. No evidence of intraparenchymal lesion or invasion to the adjacent venous sinus is noted.

Furthermore, an enhanced brain MRI was performed, which demonstrated an occipital bone-enhancing lesion with minimal intracranial soft tissue component suggestive of metastasis, with no intraparenchymal lesion in addition to non-enhancing extracranial small soft tissue component (Figures 7, 8).

**Figure 9:**
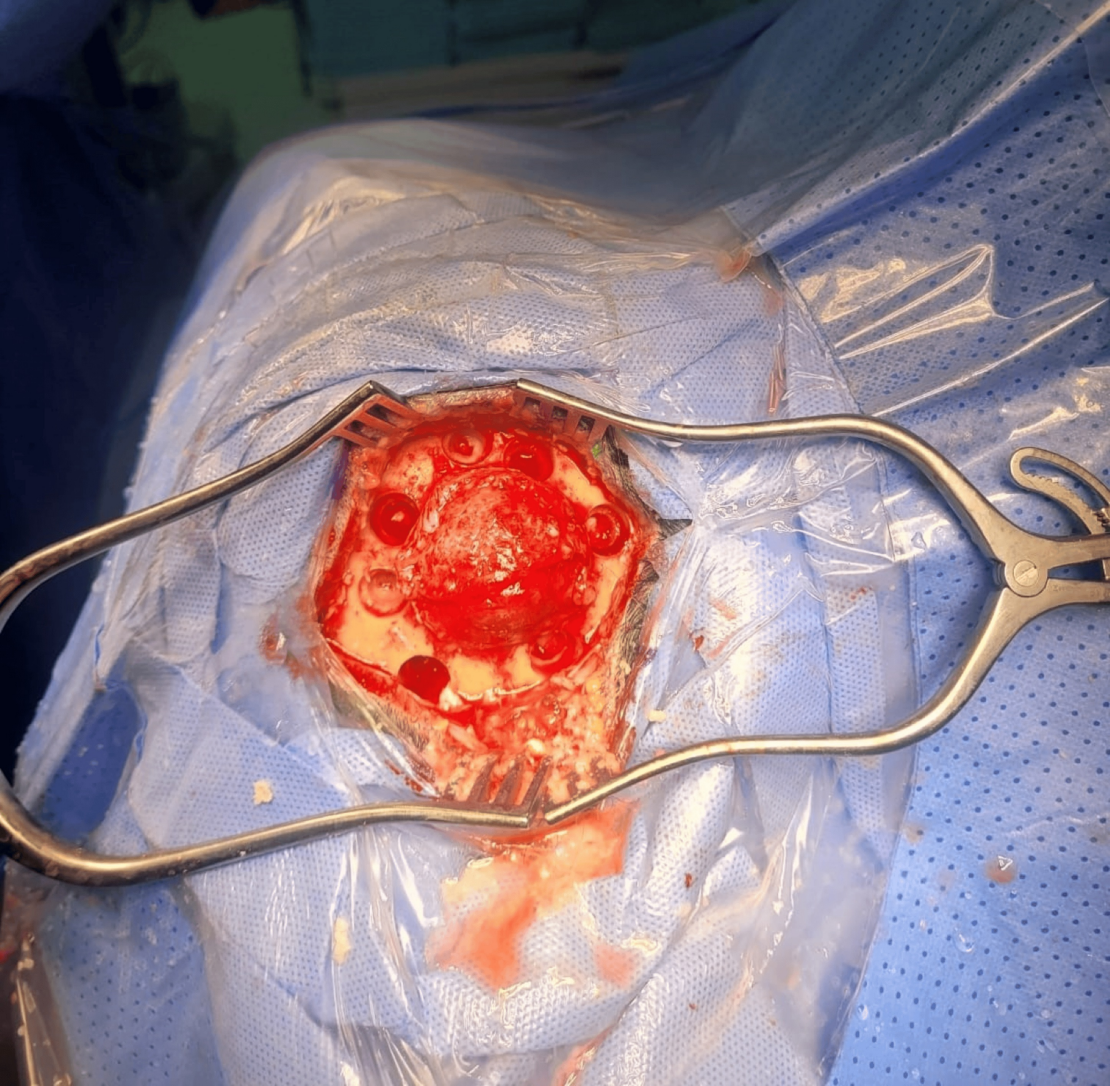
An intraoperative photograph showing the destructive skull lesion.

After the exclusion of other bone metastasis using a nuclear bone scan, the tumor board decided to perform a resection of the skull lesion (Figure 9).

**Figure 10:**
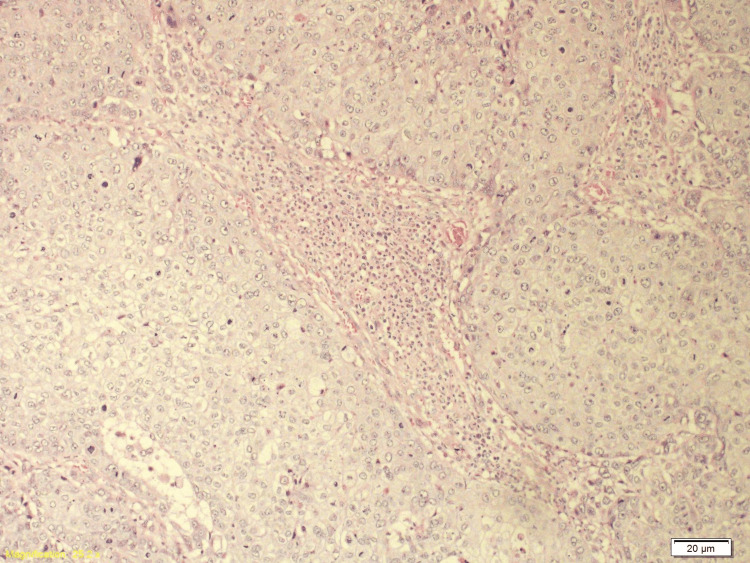
The H&E section shows a low-power view (X10) of metastatic carcinoma composed of nests and islands of squamous epithelial cells with sharp cell borders infiltrating the skull bone of the excised occipital skull lesion.

**Figure 11:**
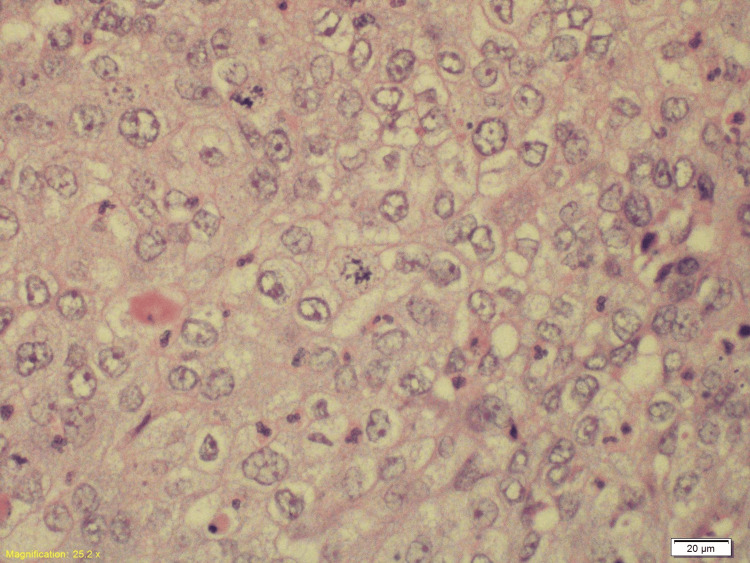
The H&E section (X40) shows squamoid cells with frequent mitoses and significant cytological atypia of the excised occipital skull lesion.

**Figure 12:**
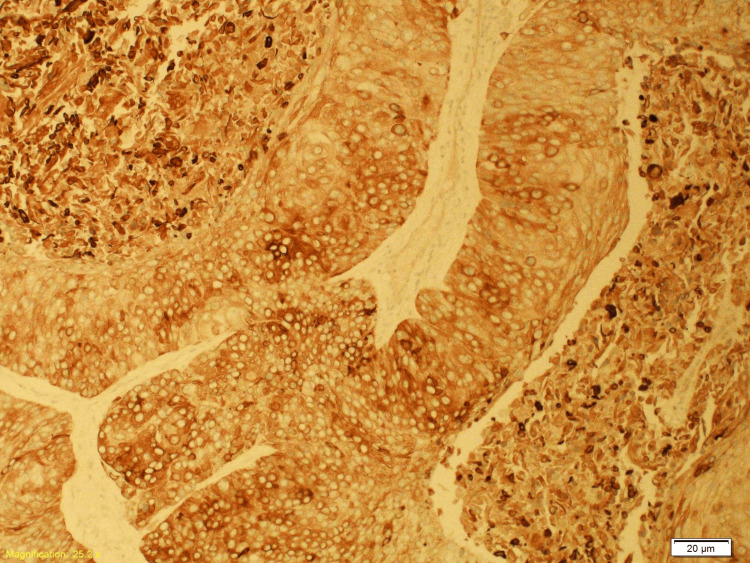
The CK5/6 immunostain is strongly and diffusely positive in the carcinomatous cells (X10) of the excised occipital skull lesion. CK 5/6: cytokeratin 5/6

**Figure 13:**
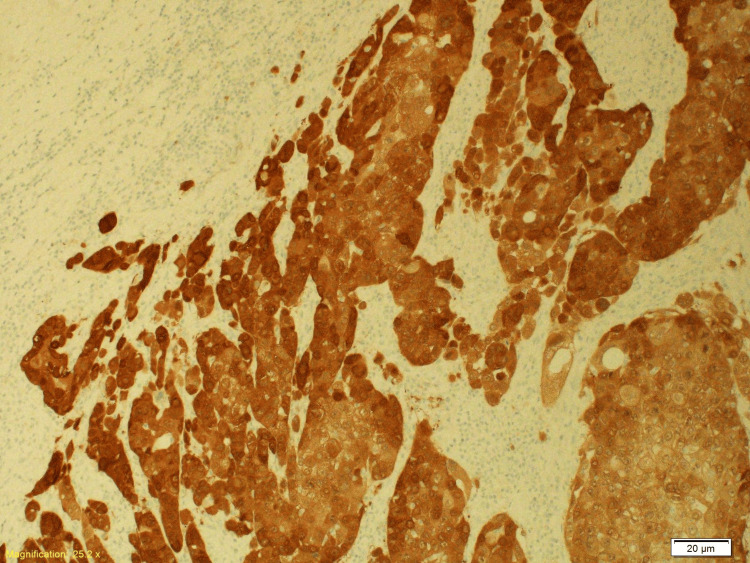
The p16 immunohistochemical stain is strongly expressed in the nuclei of the neoplastic cells, indicating HPV association (X10) of the excised occipital bone lesion. HPV: human papillomavirus

Histopathology following resection of the occipital lesion revealed metastatic HPV-associated SCC of cervical origin involving the skull bone and dura. Immunohistochemistry of the metastatic lesion was positive for programmed cell death ligand 1 (PDL-1) (Figures 10-13).

After surgery, the patient received whole brain radiotherapy of 30 Grays in 10 fractions (30 Gy/10 Fr) followed by chemotherapy (10 cycles) (cisplatin, Taxol, Avastin, and pembrolizumab) and then completed with Avastin alone due to deranged thyroid function.

## Discussion

In female patients, both breast and lung cancer represent around 80% of cancers that metastasize to bone [10], while the incidence of clinical bone metastasis in cervical cancer ranges between 1.1% and 16% [11]. Despite the low incidence of bone metastasis in cervical cancer, those patients usually suffer a poor quality of life.

It has been reported that cases of cervical adenocarcinoma, advanced stages ( FIGO stages IIB-IV), treated patients with initial multiple bone metastases, and cases of poorly differentiated tumors had less bone metastasis-free survival [12].

In cervical cancer cases, bone is a common location of metastasis following the lungs and liver [13]. It has been reported that bone metastasis from cervical cancer can occur in all FIGO stages [13]. Also, the overall median time of bone metastasis in cervical cancer patients after diagnosis was 16 months [13]. 

The mechanism of bone metastasis in cervical cancer is not well recognized. However, it could be due to the direct extension of the primary tumor to the pelvic bone, extension from nodal or soft tissue deposits to adjacent bone, or regional hematogenous or systemic hematogenous metastasis [14]. Regional hematogenous spread is thought to be through the Batson venous plexus. This valveless venous system serves as a pathway for the metastatic spread of cells to the spinal column [14].

Generally, skeletal metastases preferentially occur in the axial skeleton: vertebrae, pelvis, ribs, cranium, and the proximal appendicular skeleton. In cervical cancer, the most frequent location of bone metastasis is the spine; after that, the pelvic bones. Other cases reported isolated localized metastasis to the distal appendicular skeleton and skull [11].

Some studies stated that the cases of cervical cancer who were diagnosed with pelvic bone metastasis had an extended survival rate compared to cases with other sites of bone metastasis [13].

Symptoms of bone metastasis depend on the site of metastasis and range between localized pain, pathological fracture, spinal cord compression, hypercalcemia, nerve root compression, and neuromuscular dysfunction [15].

Skull metastasis symptoms vary according to the location of metastasis, which can be classified as calvaria, skull base, and diffuse, where each can be classified into intraosseous and invasive (dura or scalp). Skull base metastasis has worse symptoms due to cranial nerve involvement, while calvaria bone metastasis varies from no symptoms to focal pain and swelling in addition to nausea and headache once the dura is invaded [16].

In our case, the lesion involved the skull calvaria with invasion to the dura, and the patient’s presentation was an occipital bone painful lump that was increasing gradually without associated neurological symptoms.

Nowadays, PET-CT is considered an effective diagnostic modality to evaluate distant metastases with distinctive high accuracy in comparison to other radiological sectional imaging modalities [5]; however, there is a limitation in the field of view in PET-CT where the lower extremity below the mid-thigh and the skull vault is beyond the routine scan. So, we must be meticulous in selecting the metastatic work-up guided by the patient's complaint and clinical presentation, which was the scenario with our case, as she had a PET CT that revealed no metastasis, and the next day brain CT demonstrated skull metastasis.

Particularly, MRI with contrast for suspected skull metastasis is the most sensitive modality for diagnosis, localization, extension, and degree of invasion, as well as the exclusion of synchronous intraparenchymal metastasis [16]. Complementary nuclear bone scans and PET-CT are recommended to exclude other bone metastases.

Typical MRI findings in bone metastasis include the replacement of bone marrow T1 hyperintense signal by hypointense tumor signal with post-contrast enhancement [16]. The bone window of a CT skull is sensitive in demonstrating bone lesions; however, it has limitations in assessing the extension to the surrounding dura or synchronous brain metastasis compared to MRI [16].

Management of isolated skull metastasis usually includes many options such as surgical resection, radiotherapy, chemotherapy, and gamma knife surgery.

In cases of oligometastasis, combined multimodality management and vigorous local treatment have shown survival advantages and can improve the outcome [17]. Management of skull metastasis should be individualized depending on the location in the skull and symptoms of the patient, as well as the extent of the disease to other sites.

Our case underwent surgical resection after confirmation of a solitary skull lesion and the excellent response of the local disease, followed by whole-brain radiotherapy and chemotherapy.

Most cases of cervical cancer after diagnosis of bone metastasis showed declined survival time, reaching 12 months [17]. As reported, cases with hematogenous metastasis usually have a higher risk of mortality compared to cases of lymphatic metastasis, reaching up to approximately five times higher [18].

While our presented case of cervical cancer and skull metastasis tolerates the treatment well, currently has no complaints, and at the time of writing of this article, the patient has survived more than 12 months since being diagnosed with bone metastasis.

An overview of previously reported similar cases of cervical cancer with secondary skull metastasis in the literature was mentioned in an article by Tonchev et al. where three cases out of 18 were diagnosed with skull metastasis at the time of diagnosis. The initial stages of the 18 reported cases ranged between FIGO stages 1B1-IV; one case out of 18 was adenocarcinoma, while the rest of the cases were SCC and the metastasis-free periods ranged between (0 to 20 months) [19]. 

## Conclusions

This case calls attention to the necessity of not ignoring unusual symptoms in patients who have a history of cervical cancer, as hematogenous metastasis may spread at any stage to unusual sites. An MRI is the modality of choice if brain or skull metastasis is clinically suspected. Selected patients can achieve longer-term disease-free survival and better quality of life following vigorous treatment for isolated bone metastasis.

## References

[1] (2024). Cervical cancer. https://www.who.int/news-room/fact-sheets/detail/cervical-cancer.

[2] Farahat FM, Faqih NT, Alharbi RS, Mudarris RI, Alshaikh SA, Al-Jifree HM (2021). Epidemiological characteristics of cervical cancer in a tertiary care hospital, western Saudi Arabia: a retrospective record-based analysis from 2002-2018. Saudi Med J.

[3] Small W Jr, Bacon MA, Bajaj A (2017). Cervical cancer: a global health crisis. Cancer.

[4] Cohen PA, Jhingran A, Oaknin A, Denny L (2019). Cervical cancer. Lancet.

[5] Li H, Wu X, Cheng X (2016). Advances in diagnosis and treatment of metastatic cervical cancer. J Gynecol Oncol.

[6] (2025). Cervical cancer country profiles. https://www.who.int/publications/m/item/cervical-cancer-country-profiles.

[7] Pan X, Yang W, Wen Z, Li F, Tong L, Tang W (2020). Does adenocarcinoma have a worse prognosis than squamous cell carcinoma in patients with cervical cancer? A real-world study with a propensity score matching analysis. J Gynecol Oncol.

[8] (2023). Cervical cancer prognosis and survival rates. https://www.cancer.gov/types/cervical/survival.

[9] Zhou S, Peng F (2020). Patterns of metastases in cervical cancer: a population-based study. Int J Clin Exp Pathol.

[10] (2024). Metastatic bone disease. http://emedicine.medscape.com/article/1253331-overview?form=fpf..

[11] Gioe A, Arciuolo D, Carbone V (2022). Isolated humeral metastasis in cervical cancer: a case report and review of the literature. J Cancer Res Ther.

[12] Yoon A, Choi CH, Kim HJ (2013). Contributing factors for bone metastasis in uterine cervical cancer. Int J Gynecol Cancer.

[13] Thanapprapasr D, Nartthanarung A, Likittanasombut P (2010). Bone metastasis in cervical cancer patients over a 10-year period. Int J Gynecol Cancer.

[14] Ratanatharathorn V, Powers WE, Steverson N, Han I, Ahmad K, Grimm J (1994). Bone metastasis from cervical cancer. Cancer.

[15] Baliyan A, Punia S, Kundu R, Dhingra H, Aggarwal P, Garg SK (2019). Histopathological spectrum of bone changes in skeletal metastasis. Ind J Med Paediatr Oncol.

[16] Mitsuya K, Nakasu Y, Horiguchi S (2011). Metastatic skull tumors: MRI features and a new conventional classification. J Neurooncol.

[17] Zilberlicht A, Voldavsky E, Lavie O, Auslender R, Shai A (2015). Prolonged survival in a patient with isolated skull recurrence of cervical carcinoma - case report and review of the literature. Gynecol Oncol Rep.

[18] Kim K, Cho SY, Kim BJ, Kim MH, Choi SC, Ryu SY (2010). The type of metastasis is a prognostic factor in disseminated cervical cancer. J Gynecol Oncol.

[19] Tonchev N, Stein KP, Ignatov A (2022). Skull metastasis as a primary manifestation in cervical carcinoma. Ann Case Rep.

